# DNAforge: a design tool for nucleic acid wireframe nanostructures

**DOI:** 10.1093/nar/gkae367

**Published:** 2024-05-15

**Authors:** Antti Elonen, Leon Wimbes, Abdulmelik Mohammed, Pekka Orponen

**Affiliations:** Department of Computer Science, Aalto University, Finland; Department of Computer Science, Aalto University, Finland; Department of Mathematics and Computer Science, Philipps University of Marburg, Germany; Department of Biomedical Engineering, San José State University, USA; Department of Computer Science, Aalto University, Finland

## Abstract

DNAforge is an online tool that provides a unified, user-friendly interface to several recent design methods for DNA and RNA wireframe nanostructures, with the possibility of integrating additional methods into the same framework. Currently, DNAforge supports three design methods for DNA nanostructures and two for RNA nanostructures. The tool enables the design, visualisation and sequence generation for highly complex wireframe nanostructures with a simple fully automated process. DNAforge is freely accessible at https://dnaforge.org/.

## Graphical Abstract

**Graphical Abstract:**
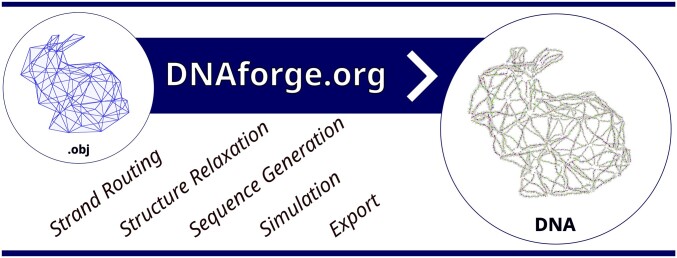

## Introduction

In nucleic acid nanotechnology, nanoscale structures are self-assembled from rationally designed strands of DNA or RNA (1,2). The base-pairing characteristics of nucleic acids make them a finely programmable fabrication material, which enables the assembly of structures with high precision and complexity, comprising currently up to tens of thousands of nucleotides.

DNA and RNA origami (3,4) are two powerful, broadly applicable design paradigms that set forth how a long baseline strand (‘scaffold strand’ in DNA origami) can be guided to fold into a desired target shape or structure by a carefully programmed array of auxiliary strands or motifs (‘staple strands’ in DNA origami, kissing-loops and other connector motifs in RNA origami). Both methods have been used to design a large variety of 2D shapes and 3D structures (5,6).

Most current 3D origami designs follow the approach of packing several 2D layers of helices or helix bundles on top of each other, and/or curving helix bundles as initially suggested in (7,8). An alternative path to 3D design is to create a *wireframe* structure that incorporates only the boundary edges and vertices of the 3D model. There have been several notable pre-origami excursions in this direction (9,10), but it has mostly started to gain a following with the development of the flexible and robust origami techniques (6,11). Some advantages of wireframe designs as compared to helix-packing ones include economy of strand use, which allows the construction of larger structures, and better folding under low-salt conditions. Some of the challenges, on the other hand, are the sometimes low rigidity of the structures, particularly for large single-helix edge designs (this can be alleviated by employing multi-helix edges, at the cost of increased strand use) and the low yield of large and complex designs.

There already exist several nucleic acid nanostructure design tools (8,12–20). Most of these however address helix-packing designs, with the more recent ones oriented towards wireframe structures including vHelix (14), DAEDALUS (21) and ATHENA (17) for 3D DNA wireframes, Sterna (19) for single-stranded 3D RNA wireframes and PyDAEDALUS (20) for 3D RNA/DNA hybrid wireframes. These tools however mostly support one specific design approach each and many are also off-line, requiring a separate process for installing the tool and its ancillary libraries, which may sometimes be difficult to locate or in the worst case deprecated.

## The DNAforge tool

DNAforge is a web application that provides a user-friendly unified interface and an extensible framework for the automated design of nucleic acid wireframe nanostructures. The tool currently covers five design methods with complementary characteristics, and is open to future extensions. It produces designs that include the full 3D nucleotide model, stapling arrangement where applicable, and the primary sequences. The designs can also be exported to the widely-used oxView tool (22,23) for further editing, visualisation and analysis, and to the oxDNA molecular dynamics engine (24–26) for simulation. DNAforge also provides an option for in-tool oxDNA simulation, if the engine and a connecting backend module are locally installed.

DNAforge is written in the TypeScript enhancement of JavaScript, converted into JavaScript ES6 and bundled with the Webpack JavaScript bundler for use in a browser. It makes significant use of the Three.js 3D rendering library and the Cannon-es 3D physics engine. The code’s dependencies on these and other libraries are managed with the package manager NPM. The DNAforge user interface is built on the same Metro4 UI library as that of oxView, which should make it easily approachable to the nucleic acid nanotechnology community.

At present, DNAforge supports five design methods: the A-trail and spanning-tree based design methods for scaffolded DNA origami from (14) and (21), respectively; a cycle cover -based method for scaffold-free DNA wireframes that extends the one presented in (27) (extension unpublished), the spanning tree -based method for RNA origami from (19) and an enhancement to it (unpublished). These methods are briefly summarised below, and we refer the reader to the original publications for the detailed descriptions. The DNAforge workflows are nearly identical for all the methods, as outlined below.

## Design workflow

**Figure 1.**
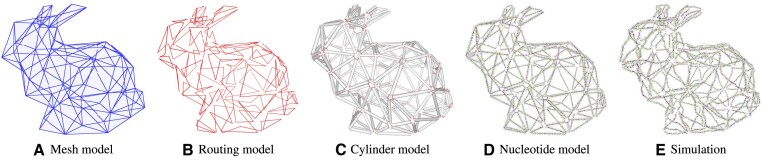
The DNAforge design workflow.

The first step in the DNAforge design workflow (Figure 1) is to create a 3D mesh model in the standard OBJ format (Figure 1A). This can be done using some 3D modelling suite such as Blender (28) or Maya (29), and a large variety of models are also available on the Internet. This input mesh is then used as the basis for one of the available design methods, each of which produces a routing model (Figure 1B), a cylinder model (Figure 1C), and a nucleotide model (Figure 1D).

The abstract routing model is produced first. It is then converted to a cylinder model, where each cylinder represents a double helix. Strain in the system can optionally be relaxed via a physical simulation that tries to minimise the length of each helix-to-helix connection, while still preventing helical overlaps. The cylinder model is then used to generate a nucleotide-level model, which fully determines the 3D structure of the designed DNA or RNA nanostructure. Finally, a primary structure can be generated. The full 3D design can be exported as a PDB file or UNF file (30) for further processing by other tools, and the strand sequences can be exported as a CSV file for ordering and synthesis.

### Routing model

The routing model represents the paths one or more strands take around the wireframe of the mesh. For the A-trail method, the model indicates a scaffold path that follows a topologically constrained Eulerian circuit traversing around a reconditioned wireframe. For the Spanning-tree/DNA, Spanning-tree/RNA, and Xuong-tree/RNA methods, it represents a single strand path that traverses twice around the spanning tree of the wireframe. For the Cycle-cover method, it comprises a number of directed cycles that cover the wireframe by traversing each edge once in both directions. The strand paths in the five routing methods currently implemented are sketched on the Schlegel diagram of a tetrahedron in Figure 4. The details of these methods are presented in the Design Methods section.

**Figure 2.**
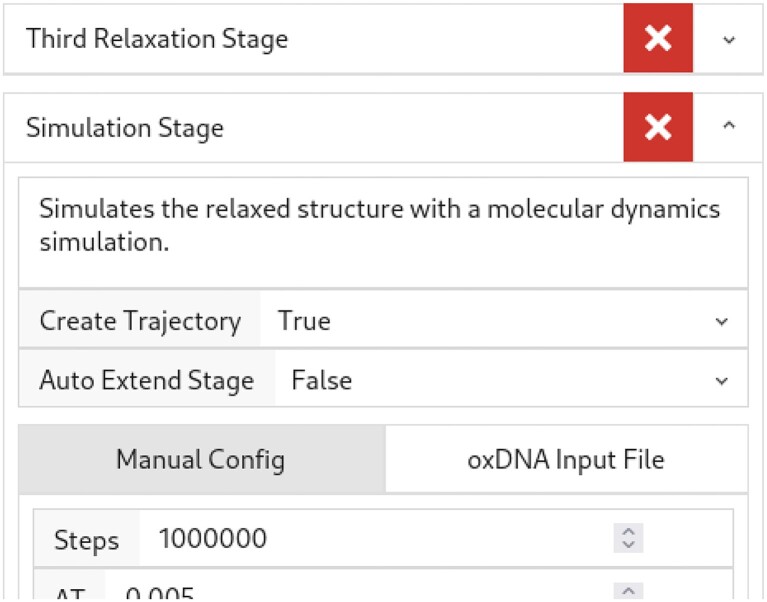
Configuring simulation jobs on DNAforge.

**Figure 3.**
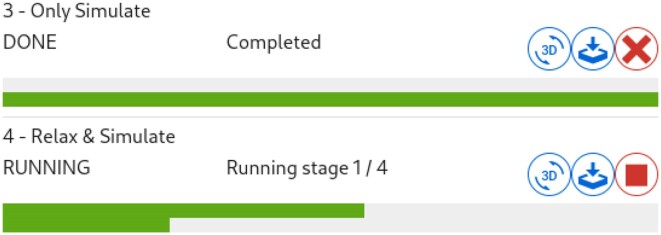
Running simulation jobs on DNAforge.

**Figure 4.**
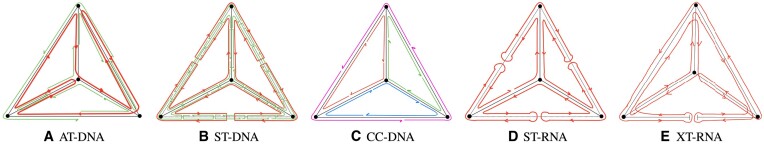
Schematics of design methods currently supported by DNAforge: A-trail/DNA (14), Spanning tree/DNA (21), Cycle-cover/DNA (27), Spanning-tree/RNA (19), Xuong-tree/RNA (unpublished).

The routing model defines how the edges of the wireframe model should connect to each other, i.e., it determines the connections of the individual cylinders of the cylinder model.

### Cylinder model

DNAforge adopts the common abstraction of a double helix as a cylinder. The cylinder model is used to calculate the exact number of nucleotides aligned along each wireframe edge and to avert physical overlaps between neighbouring helices. Each cylinder has a specific length and radius and contains four connection points representing the two 5′ and two 3′ ends of a double helix.

Cylinders are positioned and connected to each other according to the routing model. Their sizes are determined by a scale parameter provided by the user. The scale parameter converts the length units of the input mesh into nanometers. At a scale of 1 nm, an edge of length 1 would be converted to a cylinder of length 1 nm. The positions of a cylinder’s connection points and its diameter depend on the type of the cylinder model (DNA or RNA) and on the number of nucleotides aligned on it, which in turn depends on the length of the cylinder. For a DNA cylinder, the diameter is fixed at 2 nm, which corresponds to the mean diameter of a physical B-type DNA double helix. For an RNA cylinder, the diameter is set to 2.3 nm, corresponding to an A-type RNA double helix.

Technically, the scale, position, and orientation of a cylinder are represented by a 4 × 4 matrix. This allows for the nucleotides generated along the cylinder surface to be mapped to their global coordinates by simple matrix multiplication.

The cylinder model can still optionally be relaxed using a physical simulation implemented on the cannon-es physics engine. The cylinders are modelled as cylinder-shaped rigid bodies, with their connection points joined together with constrained springs. The springs try to pull neighbouring cylinders together, but collisions between the rigid bodies prevent them from overlapping. The relaxation procedure tries to minimise the lengths of the linker strands between connected cylinders, which is useful when using a variable number of linker nucleotides in the design or when exporting the nucleotide model for simulation.

### Nucleotide model

The nucleotide model is generated from the cylinder model. Each cylinder is converted into two antiparallel strands, and the strands’ 5′ and 3′ ends are connected to each other with short linker segments according to the connectivity of the cylinder model. Depending on the design method, the strands are also re-routed further: for instance, some cylinders in the ST-RNA design method (see below) should form kissing loops, which are created by changing the connectivity of certain nucleotides in the middle of the strands.

The individual nucleotides are generated and oriented by first creating a template DNA or RNA double helix along the Y-axis. The number of nucleotides is determined by the length of the cylinder in nucleotide distance units. The double helix is then rotated and positioned to match the target cylinder’s rotation and position, viz. the transformation matrix of each nucleotide is multiplied by the cylinder’s transformation matrix. The linker strands between cylinders are generated by spherical linear interpolation between the positions of the 3′ and 5′ ends of the strands connected by the linker.

Once the linker strands are added, the nucleotide model consists of long cyclic strands. Strand gaps, or nicks, can be added to these to break the cycles into shorter oligonucleotide splints (‘staple strands’ in scaffolded DNA origami), making these as short as possible without compromising binding stability. The longer the overlap between two strands, the stronger their mutual binding. However, the length of the binding domains also affects the total length of the strands, which is what the nicking procedure aims to minimise. In DNAforge, the minimum overlap is a user-defined parameter.

After the nucleotide model is generated and the nucleotides to be base-paired been identified, appropriate complementary strand sequences can be devised. For the scaffold-based design methods, the scaffold strand sequence – commonly the M13mp18 viral genome or one of its variants – determines the sequences of the staple strands. DNAforge provides a number of standard scaffold strand choices, and also the option for a user to upload a custom sequence. The ST-RNA method, on the other hand, utilises for a part of the sequence design the NUPACK tool (31), for which DNAforge provides an export function, and the Cycle-cover method has its own primary structure generator based on a local search algorithm.

The completed nucleotide model can be exported as a PDB file or as a UNF file for further visualisation, editing and simulation, and the strand sequences as a CSV file towards an eventual synthesis of the structure.

### Simulation

DNAforge also provides the possibility of in-tool simulation and visualisation by the oxDNA molecular dynamics engine (24–26). The tool comes with an optional backend that acts as a wrapper around oxDNA, exposing many of its capabilities to DNAforge via a REST API and a WebSocket. The wrapper is written in Kotlin and can either be run using the provided Docker Compose file or by installing oxDNA locally and compiling the wrapper.

Using this backend, a given nucleotide model can be simulated and visualised live directly in the DNAforge interface, as demonstrated by the snapshot of a nucleotide model in simulation in Figure 1E.

OxDNA simulations almost invariably need a pre-simulation relaxation process to get the nucleotide model into an acceptable initial conformation, lest the simulation ‘blow up’ because of nonphysically strong simulated interaction forces. This relaxation process can be quite complex, requiring several stages with different methods and durations.

By default, this relaxation process comprises three stages: a simple potential energy minimisation, a Monte Carlo simulation, and finally a molecular dynamics simulation with a very small integration time step. The more complex the structures are, the longer the individual relaxation stages need to run.

Parameters for these default stages can be easily modified via the DNAforge web interface (Figure 2) before submitting a simulation job to the queue (Figure 3) at the backend.

Perhaps most importantly, the backend also supports adaptive extension of the relaxation stages, based on potential energy reductions observed in the process. Thus the user does not need to estimate the appropriate lengths of the relaxation stages or find these by trial and error, but the backend intrinsically observes the progress of the relaxation and terminates it only when the structure seems to have reached a feasible initial conformation for the actual simulation process.

## Design methods

### A-trail/DNA (AT-DNA)

In the A-trail or ‘BScOR/vHelix’ approach to scaffolded DNA origami design (14), the scaffold strand (red curve in Figure 4A) is traced along the edges of a wireframe with minimal repetition, while simultaneously constraining the turns at every junction to be either a sharp left or a sharp right. For the notion of sharp-turns to be well-defined, all the edges of the wireframe must be incident to polygonal faces, which when glued together must form a surface (2-manifold) mesh.

The DNAforge search algorithm for A-trails employs a branch-and-bound algorithm over two possible configurations of sharp-turn choices at each junction, leading to a worst-case exponential runtime in the number of junctions. Nevertheless, the heuristics and pruning incorporated in the algorithm almost always find A-trails for triangulated wireframe structures that can be assembled from the typical ∼7 kb scaffolds.

When the polygonal mesh is a topological sphere, the sharp turn condition of A-trail routings ensures that the designed scaffold route is unknotted (32), and thus congruous with the standard, topologically unknotted circular scaffold strands. While DNAforge can also search for and typically finds A-trails for toroidal meshes, it offers no guarantees about their unknottedness (32,33). In addition to the automated A-trail search, DNAforge also supports manual import of A-trails, if for instance a user wants to use unknotted A-trails for toroidal meshes that were generated manually or using other tools.

Furthermore, selected single-helix A-trail edges can be reinforced, at the click of a button, into multihelix ones as in (34).

### Spanning-tree/DNA (ST-DNA)

The Spanning-tree or ‘DAEDALUS’ design method for DNA (21), presented in Figure 4B, routes the scaffold strand twice around a spanning tree of the input wireframe. (A spanning tree is a connected set of edges that covers every vertex.) In the ST-DNA implementation presented in (21), the spanning tree is found by Prim’s algorithm, which produces a maximally branching tree. Each spanning-tree edge is rendered as a two-helix bundle that is held together by staple crossovers. Non-spanning-tree edges are similarly realised as two-helix bundles but have scaffold crossovers between the helices. The helix lengths are rounded to an integer number of turns to facilitate the specific stapling pattern of the method. The staple sequences are set to be the Watson-Crick complements, per paired segment, to the scaffold strand sequence chosen by the user.

The linear-time ST-DNA method in DNAforge can find a routing for any reasonably sized connected wireframe quickly. The routing is also guaranteed to be an unknot, although the geometry of the double helices at the vertices might cause the nucleotide model to be knotted. Since the ST-DNA method uses two double helices for each edge, the resulting structure can be expected to be more rigid than single-duplex designs. However, as each edge consumes twice as much of the scaffold strand, the largest structures that can be designed are only about half as big as those reachable with the A-trail approach.

### Cycle-cover/DNA (CC-DNA)

The Cycle-cover method, illustrated in Figure 4C, is a scaffold-free approach where cyclic strands are arranged along a set of graph-theoretic cycles in the input wireframe model so that each edge of the wireframe gets covered twice in antiparallel directions. These double-covering cycles can be built in a bottom-up way from strand crossovers at vertices, where it is ensured that the arms of the junctions are connected. The cyclic strands are then nicked in a staggered manner to yield the desired routings of the short linear strands. The lengths of the linear strands and the extent of their overlapping regions are controlled by user-defined parameters. The method is an extension of the one presented in (27,35), which only considers cycles around the mesh model’s faces. In this extended form the method can generate scaffold-free designs for all connected wireframes.

The strand sequences in the Cycle-cover method are generated with the Focused Metropolis Search (36) algorithm. This local search algorithm tries to minimise the length of the longest repeated substring to avoid non-specific and unintended pairings while adhering to the user-supplied constraints on GC content, linker bases, and prohibited subsequences. Fully complementary non-specific pairings can exist only if repeated substrings exist, but a repeated substring does not necessarily mean that there is potential for a non-specific pairing. The distinction, however, is not very restrictive, which is why it was chosen as an optimisation target. Due to the computational complexity, only substrings contained entirely within strands are considered, rather than subsequences or substrings spanning across more than one strand.

### Spanning-tree/RNA (ST-RNA)

The Spanning-tree engineered RNA design or ‘Sterna’ method (19), presented in Figure 4D, is an RNA origami technique that first routes a single linear RNA strand twice around a spanning tree of the input wireframe, in order to form A-helices constituting the core secondary structure. It then bulges out hairpin motifs from each vertex of the wireframe towards the middle of the non-tree edges, so that each non-tree edge can eventually be rendered as a 180^○^ kissing loop pair. These kissing loops behave much like regular double helices and as such are modelled with normal cylinders in the cylinder model. The open 3′-to-5′ nick is placed at the longest wireframe edge.

The primary structure for an ST-RNA design can either be generated entirely randomly, subject to Watson-Crick complementarity conditions, or it can be generated externally and imported back into DNAforge. DNAforge has an export function which creates a NUPACK-runnable input file, where the kissing loops and certain specific bases are already set. The output of NUPACK can then be imported back into DNAforge. The kissing loop sequences are selected from a pre-generated list, which contains sequence pairs that form strong and highly specific bonds.

The DNAforge implementation of the ST-RNA method has similar limitations and guarantees as the ST-DNA method: It allows for the routing of any connected graph, and the route is guaranteed to be an unknot, but the nucleotide model can still be knotted due to the geometry of the double helices. ST-RNA can be used to generate highly complex structures with hundreds of kissing loops, but experimentally it seems that structures with more than a few dozen kissing loops have difficulties in folding correctly and/or tend to form large aggregates of nonspecifically paired particles, unless experimental conditions are very carefully controlled (19).

### Xuong-tree/RNA (XT-RNA)

The Xuong-tree method (Figure 4E) is an RNA strand routing technique building on the ST-RNA ideas that minimises the number of kissing loops in the eventual structure. It achieves this by finding for the input wireframe a specific spanning tree, a Xuong Tree, that minimises the number of odd-sized components in its co-tree, viz. its edge-complement graph (37,38). A stably bound single-stranded route can be constructed around this spanning tree and the even-sized components of the co-tree, but one kissing loop is necessary for each of the odd-sized components. For so called upper-embeddable graphs, which includes all fully triangulated meshes (39), at most a single kissing loop in total is needed. As with the ST-RNA method, the strand gap in an XT-RNA design is placed at the longest wireframe model edge. Due to the highly pseudoknotted nature of XT-RNA designs, the primary structure is currently generated randomly, subject to Watson-Crick pairing conditions.

The XT-RNA algorithm can be used on any connected graph, and the routing model can be resolved as an unknot on spherical meshes. DNAforge offers no guarantees about the unknottedness of routes on non-spherical meshes or at the level of the nucleotide model. Since structures generated with the XT-RNA method typically contain at most a single kissing loop, it can potentially be used to create more complex successfully folding and less aggregation-prone RNA structures than the ST-RNA method.

## Conclusion

We have introduced DNAforge, an online software tool for designing 3D wireframe nucleic acid nanostructures. The tool is hosted on GitHub (https://github.com/dnaforge/) under the MIT license and can be accessed at https://dnaforge.org/. This website is free and open to all users and there is no login requirement.

DNAforge does not seek to be a unified platform for all design methods or replace tools such as caDNAno, oxView and oxDNA. For instance, lattice-based designs are out of its scope, and we do not aim to extend its capabilities for nucleotide-level manipulation or directly incorporate functions that require significant computational power.

Development opportunities for DNAforge include incorporating new design methods, enhanced control of some structural elements, and providing display options for atomic-level modeling. In the longer term, we intend to provide an API for third-party developers.

## Supplementary Material

gkae367_Supplemental_Files

## Data Availability

The tool is hosted on GitHub (https://github.com/dnaforge/) under the MIT licence and can be accessed at https://dnaforge.org/. This website is free and open to all users and there is no login requirement. DOIs for the frontend and backend repositories, respectively: https://doi.org/10.5281/zenodo.11045778, https://doi.org/10.5281/zenodo.11045829.
