# Supplementary material for: DNAforge: a design tool for nucleic acid wireframe nanostructures
Source: Nucleic Acids Res. 2024 May 15;52(W1):W13–8. doi: 10.1093/nar/gkae367 (PMC11223811; doi:10.1093/nar/gkae367)
Supplement: gkae367_Supplemental_Files [file gkae367_supplemental_files.zip › supplement.pdf]

Supplementary Information

April 9, 2024

This PDF file includes:

Supplementary note 1: Technical workflow

Supplementary note 1: Technical workflow

The DNAforge design pipeline consists of the following steps: 1. Read the obj file into a graph data structure 2. Find an abstract route around the graph based on the design method 3. Create a cylinder model, whose connections are determined by the found route 4. Convert the cylinder model into a nucleotide model.

Graph

The graph data structure consists of sets of vertex-, edge-, and face-objects that each contain normal vectors and information about their neighbours. The edge-objects also contain two half-edge-objects, which facilitate easy directed traversal of the mesh.

Route

In general, a route consists of a sequential list of half edges, i.e., directed edges, or of a set of lists of half edges. These sequences are then converted to the cylinder model.

Cylinder model

The cylinder model is created by placing one or more cylinders for each edge of the graph model. The radii of cylinders are determined by the scale parameter and by the radius of a reference nucleotide double helix, B-DNA and A-RNA for DNA and RNA, respectively. Their lengths are determined by the scale parameter and possibly by additional input parameters, e.g,, a parameter minimising the size of steric zones.

Each cylinder is assigned four connection points, two 5-primes and two 3-primes. Since the scale and length of the cylinder is now defined, the number of nucleotides, N, fitting inside it can be calculated based on the reference double helix. The first 5-prime is set at one end of the cylinder, and the corresponding 3' is found by rotating the 5' by N * 'twist' and by translating it by N * 'rise'. The complementary primes are then found by rotating these by 'axis' degrees and by translating them by 'inclination'.

Finally, the connection points are linked from cylinder to cylinder based on the route, the cylinder model is relaxed so that the links are as short as possible, and the cylinder model is converted to a nucleotide model.

The cylinder data structure simply consists of scale, length, transformation matrix, reference nucleotide parameters, and of information about the routing method.

Nucleotide model

The nucleotide model is constructed by filling each cylinder with nucleotides, starting from the first 5' and proceding towards the 3' by rotating each nucleotide by 'twist' degrees and translating it by 'rise' nanometers, one nucleotide at a time. The generated strands can then be modified further based on the design method. E.g., ST-RNA reroutes and deletes certain nucleotides to convert regular double helices into kissing loops, and ST-DNA reroutes some strands to create crossovers instead of regular double helices. The link-segments between cylinders/double helices are converted into a number of spacer nucleotides based on the input parameters, and they are oriented by linear spherical interpolation based on the orientations of the nearby 5'- and 3'-nucleotides.

At this point the nucleotide model will consist of one or more cyclical strands. Depending on the design method and input parameters, the structure of the nucleotide model is left as is, or strand gaps are added to break the cycles. E.g., ST-RNA and XT-RNA add only one strand gap at the longest edges, and, for AT-DNA, one cycle is left as the scaffold strand, and the complementary strands are nicked at various locations to convert them to staple strands. For CC-DNA, both strands of each cylinder is nicked, if there is room.

Finally, the primary structure is generated by filling the scaffold strand bases, if one exists, and by assigning complementary bases for each basepair. Different methods can have different prmary structure generation algorithms.

Technically, the nucleotides correspond exactly to the reference double helix, and they are simply translated to world coordinates by multiplying their local coordinates with the transformation matrix of the associated cylinder.
