# Supplementary material for: DNAforge: a design tool for nucleic acid wireframe nanostructures
Source: Nucleic Acids Res. 2024 May 15;52(W1):W13–8. doi: 10.1093/nar/gkae367 (PMC11223811; doi:10.1093/nar/gkae367)
Supplement: gkae367_Supplemental_Files [file gkae367_supplemental_files.zip › user_guide.pdf]

DNAforge User Guide

Introduction

DNAforge is a generic design tool for DNA and RNA nanostructures based on 3D wireframe mesh models. The tool currently supports five design methods: the A-trail and spanning-tree methods for scaffolded DNA origami from 1 and 2, respectively; a cycle-cover method for scaffold-free DNA structures that generalises the one presented in 3; and the spanning-tree method for RNA origami from 4, along with a variant that minimises the number of kissing loops in the eventual structure 5.

The DNAforge design process is fully automated and managed in a graphical web interface following a simple workflow: The user first uploads a 3D mesh model in the standard OBJ format and chooses the preferred design method tab. Then a click of a button determines the appropriate routing model for the nucleic acid strands to cover the mesh. Another click generates a cylinder model of the eventual helices and their interconnections. This can be relaxed to reduce the strain in the structure, before producing the full nucleotide model with another click. For each design method, a number of parameters (choice of scaffold strand, scale of the structure, etc.) can be adjusted, and the relaxation of the cylinder model can be manually fine-tuned.

DNAforge is free and open source software. The frontend is written in TypeScript and can be run on all modern web browsers either online or locally. The nucleotide model can be exported in either PDB, UNF or oxDNA format for further manual editing, visualisation or simulation using e.g. the oxView and oxDNA tools. The collection of primary structure sequences can be exported separately as a CSV file. The nucleotide model can also be simulated directly from the interface, provided the DNAforge backend together with the oxDNA package are locally installed.

The tool can be accessed online at https://dnaforge.org.

Table of Contents

- Examples
- Workflow
  - Input
  - Routing Model
  - Cylinder Model
  - Nucleotide Model
  - Primary Structure
  - Output
- Design Methods
  - AT-DNA
  - CC-DNA
  - ST-DNA
  - ST-RNA
  - XT-RNA
- Editing
- Interface
- Simulation
- References

Examples

Step-by-step examples can be found here.

Workflow

This is an overview of the DNAforge design workflow. For a more detailed description of the parameters of individual design methods, see Design Methods.

Input

The first step of using the DNAforge tool is to select and load a 3D-model in the OBJ-format. It is then used as a basis for one of the available design methods, each of which produces a routing model, a cylinder model, and a nucleotide model. OBJ-files can be opened by clicking the open-button in the File-tab or by simply dragging and dropping a file into the 3D viewport. DNAforge also includes some built-in 3D models, which can be opened by clicking the Example-button.

Routing Model

The routing model represents the path one or more strands take around the wireframe mesh. The routing model defines how the edges of the wireframe model should connect to each other, i.e., it determines the connections of the individual cylinders of the cylinder model. Each design method has its own routing model. The routing model is automatically generated when necessary, but it can also be explicitly created by clicking the "Generate"-button in the desired design method tab.

Cylinder Model

The cylinder model is a simple abstraction for double helices that allows DNAforge to calculate the exact number of nucleotides corresponding to an edge and fit them in a way that prevents overlaps. Each cylinder has a certain length and radius and four connection points corresponding to the two 5-primes and two 3-primes of a double helix. A cylinder model is created automatically when necessary if one does not already exist, but it can also be created by clicking the "Generate Cylinders"-button in the desired design method tab. If the scale-parameter is changed, the cylinder model must also be recreated to reflect the change.

Relaxation

The cylinder model can optionally be relaxed using a physical simulation by clicking the "Relax Cylinder Model"-button in a design method tab. The cylinders are modelled as cylinder-shaped rigid bodies, and their primes are connected together with spring constraints. The springs try to pull neighbouring cylinders together, but collisions between the rigid bodies prevent them from overlapping. The relaxation procedure tries to minimise the lengths of the spacer strands between cylinders, which is useful when using a variable number of spacer nucleotides or when exporting the nucleotide model for simulation.

Nucleotide Model

The nucleotide model is generated based on the cylinder model by clicking the "Generate Nucleotide Model"-button. Each cylinder is converted into two antiparallel strands, and their 5'- and 3'-ends are connected to each other with short spacer segments according to the connectivity of the cylinder model. Depending on the design method, the strands are also re-routed further: For instance, some cylinders in Sterna should form kissing loops, which are created by changing the connectivity of certain nucleotides in the middle of the strands.

Primary Structure

Once the nucleotide model is generated, it can be assigned a primary sequence. For scaffold-based design methods, the primary sequence is automatically assigned based on the scaffold chosen from the drop-down menu. For further information about the procedures for the different design methods, see the appropriate entries in Design Methods.

Output

The nucleotide model can be exported as a UNF-file, as oxDNA-files, or as a PDB-file, by clicking the "Download"-buttons. The primary sequence can be exported into a CSV-file as well.

Design Methods

AT-DNA

The A-trail routing, AT-DNA, follows the procedure laid out by Benson et al. It uses a breadth-first branch and bound algorithm over two node types, left- or right-handed, to find a connected graph, resulting in an A-trail. Note: This algorithm has a high time complexity, and DNAforge is unlikely to find a routing for meshes larger than about 100 nodes.

Reinforcement

Cylinders can be reinforced by clicking the "Reinforce"-button. This will replace the selected cylinder with four-cylinder bundles. Based on the procedure described by Högberg et al.

Custom Route

A custom routing can be entered by clicking the "Upload Route"-button. The route must be a list of node ID's separated by spaces. Node ID visibility can be toggled in the interface menu.

Parameters

- **Scale** Input scale in nanometers. This is the side length of each grid square.
- **Scaffold** The primary structure of the scaffold strand.
- **Add Staple Breakpoints** Add breakpoints to convert complement strand(s) into individual staples. If off, the complement strands will form cycles.

Additional Parameters

- Routing Parameters
  - **Checkerboard reconditionin** Guarantee unknotted scaffold routing for tori by doubling additional edges to make the mesh checkerboard colourable. (Mohammed 2020).
  - **Minimise steric zones at nodes** Bring cylinders as close to the node as possible. Otherwise keeps each cylinder equally distant from the node.
  - **Search time** Stop the search after this many seconds
- Strand Parameters
  - **Midpoint nicking** Place the strand gaps at the midpoint of each edge.
  - **Max Staple Length** Maximum staple length in nucleotides.
  - **Min Binding Length** Minimum length of a continuous double helix without strand gaps.
  - **Min Spacer Nucleotides** Minimum number of spacer nucleotides generated between each stem segment.
  - **Max Spacer Nucleotides** Maximum number of spacer nucleotides generated between each stem segment.
  - **Offset** Offset for the scaffold. Ignore this many bases from scaffold start.
  - **5' ID** ID of the first nucleotide in the scaffold strand.
- Relaxation Parameters
  - **Edge constraints Constraints** Add springs between the mesh edges and cylinders.
  - **Bundle Constraints** Add springs between cylinders in bundles, e.g. reinforced cylinders or doubled edges.
  - **Spring Constraints** Add springs between 3'- and 5'-primes of neighbouring cylinders.

CC-DNA

Cycle cover, CC-DNA, is based on the scaffold-free routing method by Wang et al., where the long scaffold strand is done away with and replaced with a number of shorter staple-strands. Cycle cover routing is done greedily node by node. Each incoming edge is first split into two, corresponding to the two antiparallel strands of a double helix, and they are then connected to each other in such an order that no edge is visited twice until all edges have been visited at least once. This will result in a cycle cover over the entire mesh, allowing any connected wireframe to be routed.

Primary structure

The primary structure is generated with Focused Metropolis Search, a local search algorithm. The search algorithm tries to minimise the length of the longest repeated substring to avoid non-specific and unintended pairings while adhering to the user-supplied constraints of GC-content, spacer-bases, and prohibited subsequences. To generate a primary structure, click the "Generate Primary Structure"-button. Parameters controlling the generator can be found in the additional settings.

Parameters

- **Scale** Input scale in nanometers. This is the side length of each grid square.
- **Add Staple Breakpoints** Add breakpoints to convert complement strand(s) into individual staples. If off, the complement strands will form cycles.

Additional Parameters

- Routing Parameters
  - **Minimise steric zones at nodes** Bring cylinders as close to the node as possible. Otherwise keeps each cylinder equally distant from the node.
  - **Max Cycles** Maximise the number of cycles / minimise the embedding genus.
  - **Min Cycles** Minimise the number of cycles / maximise the embedding genus.
  - **Min Spacers** Ignore genus and try to minimise the lengths of spacer segments.
- Strand Parameters
  - **Max Strand Length** Maximum strand length in nucleotides.
  - **Min Binding Length** Minimum length of overlapping segments in nucleotides.
  - **Min Spacer Nucleotides** Minimum number of spacer nucleotides generated between each stem segment.
  - **Max Spacer Nucleotides** Maximum number of spacer nucleotides generated between each stem segment.
- Primary Structure Parameters
  - **GC-content** Target Proportion of G's and C's in the generated primary structure.
  - **Spacer Nucleotides** Spacer bases in IUPAC notation. Separated by spaces.
  - **Forbidden Subsequences** Banned subsequences in IUPAC notation. Separated by spaces.
- Primary Generator Parameters
  - **Iterations** The generator will run for this many iterations.
  - **Trials** Base Pair changes per iteration.
  - **Eta** Acceptance probability of less favourable changes.
- Relaxation Parameters
  - **Edge Constraints** Add springs between the mesh edges and cylinders.
  - **Bundle Constraints** Add springs between cylinders in bundles, e.g. reinforced cylinders or doubled edges.
  - **Spring Constraints** Add springs between 3'- and 5'-primes of neighbouring cylinders.

ST-DNA

The spanning-tree method, Daedalus, routes the scaffold strand twice around the maximally branching spanning tree of the input mesh. The spanning tree is found by Prim's algorithm, and each edge is associated with bundles consisting of two cylinders and two double helices. The cylinder lengths are rounded to the nearest full-length turn to facilitate the specific stapling-pattern of the method.

Parameters

- **Scale** Input scale in nanometers. This is the side length of each grid square.
- **Scaffold** The primary structure of the scaffold strand.
- **Add Staple Breakpoints** Add breakpoints to convert complement strand(s) into individual staples. If off, the complement strands will form cycles.

Additional Parameters

- Routing Parameters
  - **Minimise steric zones at nodes** Bring cylinders as close to the node as possible. Otherwise keeps each cylinder equally distant from the node.
- Strand Parameters
  - **Offset** Offset for the scaffold. Ignore this many bases from scaffold start.
  - **5' ID** ID of the first nucleotide in the scaffold strand.
- Relaxation Parameters
  - **Edge Constraints** Add springs between the mesh egdes and cylinders.
  - **Bundle Constraints** Add springs between cylinders in bundles, e.g. reinforced cylinders or doubled edges.
  - **Spring Constraints** Add springs between 3'- and 5'-primes of neighbouring cylinders.

ST-RNA

Sterna routes a path around a random spanning-tree, but it utilises no staples. Instead, it routes a single RNA strand around the spanning tree, and it replaces the non-spanning-tree edges with kissing loops. The kissing loops behave much like regular double helices and as such are modelled with normal cylinders in the cylinder model.

Primary Structure

The primary structure for a Sterna design can either be generated entirely randomly, or it can be generated externally and imported back into the DNAforge tool. The DNAforge tool has an export function, which creates a NUPACK-runnable input file, where the kissing loops and certain specific bases are already set. The output of NUPACK can then be imported back into the DNAforge tool.

"Generate Partial"-button will assign bases to each kissing loop from a predefined list of strong kissing loops. It will also assign IUPAC-bases to spacer segments and periodically along the double helices in order to prevent the DNA primer from having unwanted secondary structure. "Generate Random"-button will assign random complementary bases to each yet unassigned nucleotide. "Download NP"-button generates a NUPACK input file, which can be used to generate the primary structure. "Upload Priamry"-buttons opens a dialog, where the user can manually input a primary structure.

Parameters

- **Scale** Input scale in nanometers. This is the side length of each grid square.
- **Add a Strand Breakpoint** Adds a strand gap between 5'- and 3'- ends of the strand. Otherwise the strand will form a cycle.

Additional Parameters

- Routing Parameters
  - **Minimis steric zones at nodes** Bring cylinders as close to the node as possible. Otherwise keeps each cylinder equally distant from the node.
- Strand Parameters
  - **Min Spacer Nucleotides** Minimum number of spacer nucleotides generated between each stem segment.
  - **Max Spacer Nucleotides** Maximum number of spacer nucleotides generated between each stem segment.
- Relaxation Parameters
  - **Edge Constraints** Add springs between the mesh edges and cylinders.
  - **Bundle Constraints** Add springs between cylinders in bundles, e.g. reinforced cylinders or doubled edges.
  - **Spring Constraints** Add springs between 3'- and 5'-primes of neighbouring cylinders.

XT-RNA

XT-RNA is a single-stranded RNA routing method that minimises the number of kissing loops. WIP

Primary Structure

The primary structure for an XT-RNA design can either be generated entirely randomly, or it can be generated externally and imported back into the DNAforge tool.

"Generate Partial"-button will assign bases to each kissing loop from a predefined list of strong kissing loops. It will also assign IUPAC-bases to spacer segments and periodically along the double helices in order to prevent the DNA primer from having unwanted secondary structure. "Generate Random"-button will assign random complementary bases to each yet unassigned nucleotide. "Upload Priamry"-buttons opens a dialog, where the user can manually input a primary structure.

Parameters

- **Scale** Input scale in nanometers. This is the side length of each grid square.
- **Add a Strand Breakpoint** Adds a strand gap between 5'- and 3'- ends of the strand. Otherwise the strand will form a cycle.

Additional Parameters

- Routing Parameters
  - **Minimise steric zones at nodes** Bring cylinders as close to the node as possible. Otherwise keeps each cylinder equally distant from the node.
- Strand Parameters
  - **Min Spacer Nucleotides** Minimum number of spacer nucleotides generated between each stem segment.
  - **Max Spacer Nucleotides** Maximum number of spacer nucleotides generated between each stem segment.
- Relaxation Parameters
  - **Edge Constraints** Add springs between the mesh edges and cylinders.
  - **Bundle Constraints** Add springs between cylinders in bundles, e.g. reinforced cylinders or doubled edges.
  - **Spring Constraints** Add springs between 3'- and 5'-primes of neighbouring cylinders.

Editing

DNAforge supports a handful of simple edit operations for the Cylinder and Nucleotide models. Individual elements can be selected by left-clicking them, and they can be scaled, rotated, and translated. DNAforge also supports undo- and redo- operations with ctrl+z and ctrl+shift+z.

Scale

Selected cylinders can be scaled by pressing the "S"-key. The scale can be defined by moving the mouse cursor or by entering a value with the keyboard. Left click confirms the change, and right click cancels it.

Rotation

Selected cylinders and nucleotides can be rotated by pressing the "R"-key. By default, the rotation axis is the along the camera view direction, but this can be changed by pressing x, y, or z. A single press toggles rotation along a global axis, and two presses toggles rotation along the local axes. The rotation magnitude can be defined by moving the mouse cursor or by entering a value with the keyboard. Left click confirms the change, and right click cancels it.

Translation

Selected cylinders and nucleotides can be translated by pressing the "G"-key. By default, the translation axes are orthogonal to the camera view direction, but they can be changed by pressing x, y, or z. A single press toggles the translation along a global axis, and two presses toggles translation along the local axes. The translation magnitude can be defined by moving the mouse cursor.

Interface

DNAforge viewport look and behaviour can be modified from the interface menu.

- **Selection mode** This switch determines how to handle selection by left-clicking a selectable element.
    - **None** Never select anything.
    - **Single** Select only the element clicked.
    - **Limited** Select the clicked element and all connected elements of the same type.
    - **Connected** Select the clicked element and all connected elements.
    - **Hover** Highlight elements under the mouse cursor.
- **Lights**
    - **Camera Light** Toggle a light that follows the camera.
    - **Ambient Light** Toggle a static light that illuminates the whole scene.
    - **Fog** Toggle a fog that hides elements far from the camera.
    - **Colours** Open a colour managment interface for assigning and adding new colours to viewport objects.
- **Scene**
    - **Axes** Toggle the display of global XYZ-axes.
    - **Grid** Toggle the display of the grid.
    - **Colours** Open an editor for defining custom colours for the viewport elements.
- **Graph**
    - **Solid** Toggle the display of mesh faces.
    - **Wireframe** Toggle the dispaly of mesh wireframe model.
    - **Bounding Box** Toggle the display of a 10 by 10 by 10 box around the mesh.
    - **Indices** Toggle the display of node indices.
- **Cylinders**
    - **Torque Overlay** Shade cylinders by the torque exerted by the neighbouring cylinders.
    - **Tension Overlay** Shade cylinders by the tension exerted by the neighbouring cylinders.
- **Nucleotides**
    - **Draw Backbones** Toggle the display of nucleotide backbones.
    - **Draw Bases** Toggle the display of nucleotide bases.
    - **Strands** Open a strand selection menu.
    - **Arcs** View an arc diagram of the nucleotide model.
- **Camera**
    - **Reset Camera** Reset the camera to its original state.
    - **Ortographic/Perspective** Toggle between orthogonal and perspective cameras.

Simulation

The nucleotide model can be simulated directly from the interface, provided the DNAforge backend together with the oxDNA package are locally installed. The simulation backend and its installation instructions can be found here.

References

[1] Benson, E. et al. DNA rendering of polyhedral meshes at the nanoscale. Nature 523, 441-444 (2015). DOI: https://doi.org/10.1038/nature14586

[2] Veneziano, R. et al. Designer nanoscale DNA assemblies programmed from the top down. Science 352, 1534-1534 (2016). DOI: https://doi.org/10.1126/science.aaf4388

[3] Wang, W. et al. Complex wireframe DNA nanostructures from simple building blocks. Nature Communications 10, 1067 (2019). DOI: https://doi.org/10.1038/s41467-019-08647-7

[4] Elonen, A. et al. Algorithmic design of 3D wireframe RNA polyhedra. ACS Nano 16, 16608-16616 (2022). DOI: https://doi.org/10.1021/acsnano.2c06035

[5] Elonen, A. and Orponen, P. RNA origami with minimum number of kissing loops. In preparation.
